# Quality of Life and Symptoms of Hospitalized Hematological Cancer Patients

**DOI:** 10.3390/curroncol31110494

**Published:** 2024-10-28

**Authors:** Theocharis I. Konstantinidis, Ioanna Tsatsou, Eleftheria Tsagkaraki, Evgenia Chasouraki, Maria Saridi, Theodoula Adamakidou, Ourania Govina

**Affiliations:** 1 Department of Nursing, Hellenic Mediterranean University, 71410 Heraklion, Crete, Greece; harriskon@hmu.gr; 2 One Day Clinic, Hellenic Airforce General Hospital, 11523 Athens, Greece; itsatsou@uniwa.gr; 3 Department of Nursing, Venizeleio General Hospital of Heraklion, 71409 Heraklion, Crete, Greece; ritsatsagk@gmail.com; 4 Department of Nursing, University General Hospital of Heraklion, 71500 Heraklion, Crete, Greece; exasouraki@hotmail.com; 5 Department of Nursing, University of Thessaly, 41500 Larissa, Greece; sarmar32@windowslive.com; 6 Department of Nursing, University of West Attica, 12243 Egaleo, Greece; thadam@uniwa.gr

**Keywords:** quality of life, hematological cancer patient, hematological malignancies, symptoms, fatigue, MDASI

## Abstract

Patients with hematological malignancies undergo intensive treatment and prolonged hospitalization, thus having a variety of physical and psychosocial symptoms and worse quality of life (QOL). This study aimed to assess the QOL and investigate the symptoms of hospitalized hematological cancer patients. A cross-sectional study was conducted in the hematology clinics and day units of two general hospitals of Heraklion, Crete. Adult patients with hematological malignancy and an adequate understanding of the Greek language participated. A demographic questionnaire, the European Organization for Research and Treatment for Cancer quality assessment questionnaire (EORTC QLQ-C30), and the MD Anderson Symptom Inventory (MDASI) were used. The sample consisted of 120 patients—42.5% were women, with a mean age of 65.6 years. The mean time from diagnosis was 33 months. The global health status of QoL had an average value of 47.1. The highest levels of QOL were found in the subscale of cognitive function (72.8) and the lowest in the role function (46.1). For the EORTC QLQ-C30 symptoms scale, the lowest score was found in nausea-vomiting (11.0) and the highest in fatigue (59.1). In the MDASI, in part I (core symptoms), higher levels but also medium intensities were reported at fatigue (78.3%, mean 3.5), drowsiness (65.0, mean 3.3), and distress (65.8%, mean 2.8). In part II, enjoyment of life (85.8%, mean 5.1) had the highest, and relation with other people (67.5%, mean 3.7) had the lowest scores. The increase in the severity of the core symptoms (part I) was related to females (rho = 0.193, *p* <0.05) and comorbidities (rho = 0.220, *p* < 0.05). It was also associated with a significant decrease in all functional domains and increased fatigue (rho = 0.571, *p* < 0.05) in the EORTC QLQ-C30 questionnaire. The increased global health status was related to males (rho = −0.185, *p* < 0.05) and physical functioning with younger age (rho = −0.331, *p* < 0.05), higher education (rho = 0.239, *p* < 0.05), fewer months from diagnosis (rho = −0.199, *p* < 0.05), and low comorbidity (rho = −0.209, *p* < 0.05). Finally, multiple linear regression analysis revealed that the total average symptom score of the MDASI was the most significant factor influencing the global health status of the EORTC QLQ-C30 (β = −4.91, *p* < 0.001). The increased global health status of the EORTC QLQ C30 was not significantly related (*p* > 0.05) to the social characteristics of the patients, such as education or employment, which requires further validation. The QoL of hematological cancer patients significantly decreases during treatments due to a considerable number of symptoms that must be taken into consideration for high-quality, individualized care.

## 1. Introduction

The World Health Organization defines quality of life (QoL) as “an individual’s perception of their position in life in the context of the culture and value systems in which they live and in relation to their goals, expectations, standards and concerns” [1]. It is a broad concept influenced in a complex way by physical health, psychological state, level of independence, social relations, and reactions to important characteristics of people’s environment. Quality of life is also called “subjective well-being”. The multidimensionality of QoL refers to the coverage of a broad range of content, including physical, functional, emotional, and social well-being [2]. Ultimately, QoL is a multidimensional and subjective concept interpreted and defined by each individual, reflecting different approaches to the topic [2,3].

QoL for a person with a chronic illness such as cancer is a matter of particular value because the disease itself, combined with treatment toxicity, causes significant burden and distress to cancer patients and their families [4]. Hematological malignancies are a heterogeneous disease group that requires aggressive, urgent, prolonged, and demanding treatment. Patients with hematological malignancies are chronic patients who face significant physical problems in their daily living, social interactions, and mental balance due to anticancer therapies, frequent hospitalizations, and transfusions [5]. Several features of hematological malignancies’ etiology and management result in distinctive needs and symptoms compared to solid tumor cancer patients [6]. The treatment modalities of acute hematological diseases (e.g., acute leukemia), such as hematopoietic stem cell transplantation and high-dose chemotherapy, lead to many devastating adverse events, and sometimes, despite aggressive treatment, these malignancies have rapid development and poor prognosis. On the other hand, chronic diseases (e.g., chronic leukemia) demand life-long monitoring and treatments and have subsequent side effects that also reduce QoL in all of its aspects, including physical and psychosocial [7]. These factors, combined with many treatment toxicities and adverse events, also provoke severe distress and psychological burden, leading to a worsening of QoL [8]. The observed poor QoL exists regardless of the type and stage of the disease and the type of treatment [5]. So, the clinical trajectories of hematological malignancies are variable and often unpredictable, and individualized care is mandatory but not always being delivered in clinical practice [7].

The disease and treatment effects also induce various symptoms and problems that lead to high levels of unmet supportive care needs among hematological cancer patients. Regarding physical needs, fatigue and memory loss are the most common. Prevalent needs are mainly informational, followed by psychological/emotional and physical needs [9]. Unmet supportive care needs and difficult-to-treat symptoms cause deterioration in the QoL of hematological cancer patients.

The assessment of QoL through symptoms experienced by patients with hematological malignancies is a useful indicator for designing or selecting appropriate interventions for each patient and adjusting the treatment protocols according to the arising problems and needs. Timely and individualized intervention reduces the prolonged hospital stay, alleviates these symptoms, and improves the patient’s overall well-being and the adverse effects on QoL [10].

Nowadays, after years of research, patients’ QoL is being assessed using Patient-Reported Outcome Measures (PROMs), meaning measurement tools that assess patients’ experiences of cancer care. QoL assessment is of major importance in hemato-oncology care, and the wider adoption of its measurement is needed [11]. Moreover, symptom monitoring through the use of PROMs gives an evidence-based approach to detecting symptoms reported by patients themselves, which can offer valuable information to healthcare professionals, thus improving clinical management [12].

Τhe present study aimed to assess QoL and the reported symptoms of hospitalized hematological cancer patients and investigate related factors [13].

## 2. Materials and Methods

### 2.1. Setting and Sample

A cross-sectional study was performed using a convenience sample of 120 hospitalized patients treated in the hematology department and the hematology day unit of two general hospitals in Heraklion, Crete, Greece.

The sample included patients diagnosed with hematological malignancy who were over 18 years old and could understand, read, and write in Greek. Patients who were hospitalized at the diagnosis stage of their disease and patients who had a severe mental illness or dementia and were unable to complete the questionnaires were excluded from the sample.

The patients were approached in the departments where they were treated after prior consultation with a physician or nurse. If they met the criteria and agreed to participate in the study, they filled in the questionnaires.

### 2.2. Instrumentation

Patients completed a set of questionnaires that included sociodemographic and clinical characteristics (gender, age, marital status, education level, living with, employment, residence, time from diagnosis, comorbidities), the European Organization for Research and Treatment of Cancer (EORTC), Core Quality of Life Questionnaire (QLQ-C30, version 3.0), and the MD Anderson Symptom Inventory (MDASI).

The EORTC QLQ-C30 v.3.0 instrument assesses self-reported QoL in cancer patients [14]. It consists of 30 questions divided into five functional scales (physical, role, emotional, cognitive, and social functioning), three symptom scales (fatigue, pain, and nausea/vomiting), and an overall health and QoL scale. The questionnaire also presents six single items: dyspnea, insomnia, appetite loss, constipation, diarrhea, and financial difficulties. Patients assessed their functional level and symptom severity on a Likert scale (1 = not at all to 4 = very high) and their global health status/QoL on a Likert scale ranging from 1 = very poor to 7 = excellent. Scale scores were linearly transformed into scores ranging from 0–100, so they are calculated from 0 to 100. Higher scores mean better functionality and quality of life for the functional subscales and overall health and QoL. For symptom subscales, a higher score indicates more severe symptoms. The Cronbach’s alpha coefficient for this study was 0.968. The validation of the questionnaire in Greek revealed a valid and reliable tool [15]. The Cronbach for the study was 0.894.

The MDASI is used to assess the presence and severity of cancer-related symptoms experienced by patients with cancer and their impact on daily living in the last 24 h. The questionnaire consists of two parts. The core MDASI (part I) consists of 13 symptom items and is rated based on their presence and severity. Each symptom is rated on an 11-point numeric scale ranging from 0 (not present) to 10 (as bad as you can imagine). In part II of the questionnaire, patients rate how symptoms interfere with their daily living (general activity, mood, work, relationships with others, walking, and enjoyment of life). These range from 0 (did not interfere) to 10 (interfered completely). The Greek version of MDASI was translated and validated by Mystakidou et al. (2004) [16].

### 2.3. Statistical Analysis

Data analysis was performed using the SPSS statistical program (IBM Corp. Released 2019, IBM SPSS Statistics for Windows, v.26.0, Armonk, NY, USA: IBM Corp.). The significance level was set at *p* = 0.05.

The frequency distributions of the patients’ descriptive and clinical characteristics were initially calculated. The measurement score distributions of the EORTC QLQ-C30 and MDASI were tested using the Blom method (QQplot), while the reliability coefficients were calculated on a case-by-case basis using the Cronbach method. Due to asymmetry in most subscales of the two scales, the non-parametric Spearman correlation method was used, while the Kruskal–Wallis method was used to compare their scores.

Finally, multiple linear regression analysis was conducted with the EORTC QLQ-C30 (Global Health Status) total score and the characteristics of the patients and MDASI using parameters that showed significant relationships based on univariate correlations.

### 2.4. Ethics

The research was carried out after permission from the hospitals’ ethics and research committees. Patients were informed verbally and in writing, and they signed an informed consent form before their participation in the study. In addition, the anonymous completion of questionnaires and code assignments ensured the participants’ personal data protection. Additional clinical data from their medical records were obtained after relevant permission.

## 3. Results

### 3.1. Patients’ Characteristics

Table 1 presents the demographic and clinical data of the 120 patients. Their mean age was 65.6 years (±17.5), 42.5% were women, 45.8% were between 60 and79 years, 68.3% were married or cohabiting, 42.5% had received primary education, 16.7% lived alone, and 71.7% lived in an urban area. The mean time from diagnosis was 33 months (1–276), and 36.7% of the sample had comorbidities.

### 3.2. Descriptive Data of Variables

The global health status/QoL of the EORTC QLQ-C30 indicated a mean score of 47.1, or 50% of patients had levels up to 50.0 (moderate or low levels of global health status/QoL).

The highest mean score on the functioning scale was found for cognitive functioning (72.8) and the lowest for role functioning (46.1). Among the symptoms of QLQ-C30, a lower score was found in nausea-vomiting (11.0) and the highest score (meaning the worst symptom) in fatigue (59.1) and dyspnea (44.7) (Table 2).

The top five reported symptoms by patients were fatigue, drowsiness, distress, dry mouth, and sadness, and the five least reported were memory problems, shortness of breath, nausea, diarrhea, and vomiting (Table 3). From the core symptoms (part I) of the MDASI, fatigue was reported at a higher frequency (78.3%) but also at moderate intensity (3.5 on a scale of 0 to 10), followed by drowsiness (65% and 3.3), while vomiting had the lowest frequency (15.8% and 0.6). In part II of the MDASI, enjoyment of life (85.8% and 5.1) and walking activity (82.5% and 5.1) had the highest frequency but also medium intensity.

Moreover, Table 4 presents the overall scores of symptom intensity of the MDASI. It was found that part II, which shows the interference of symptoms with the patient’s daily living, had a significantly higher average intensity score than part I of the MDASI (*p* < 0.001). However, the total average symptom score was 2.8.

### 3.3. Correlations Between QOL and Patients’ Symptoms

Table 5 presents the univariate correlations of the scores of the EORTC QLQ-C30 and the MDASI. Significant correlations were observed between the subscales of the two scales in almost all the analyses. Specifically, an increase in the symptom intensity was related to a decrease in the functionality scales of QOL (negative correlations, *p* < 0.05 and an increase in the symptoms of QOL and, therefore, a worsened QOL (positive correlations, *p* < 0.05). For example, it was observed that an increase in the symptom intensity of part I of MDASI was related to a decrease in the cognitive functional scale of the EORTC QLQ-C30 (rho = −0.600, *p* < 0.05) and an increase in fatigue in the EORTC QLQ-C30 (rho = 0.571, *p* < 0.05).

Moreover, multiple linear regression was conducted between the global health status of the EORTC QLQ-C30 and the total average symptom score of the MDASI (Table 6). Increased global health status was significantly correlated with fewer symptoms, as determined by the total average symptom score (β = −4.91, *p* < 0.001).

### 3.4. Correlations Between QOL and Patients’ Characteristics

As shown in Table 7, there are sporadic significant correlations between QOL and patients’ characteristics. For example, increased Global health status/QoL was related to the male sex (rho = −0.185, *p* < 0.05), while the physical functioning scale was related to younger age (rho = −0.331, *p* < 0.05), higher education (rho = 0.239, *p* < 0.05), fewest months after diagnosis (rho = −0.199, *p* < 0.05), and fewer concomitant diseases (rho = −0.209, *p* < 0.05). Regarding the symptoms items, fatigue was associated with older age (rho = 0.277, *p* < 0.05); nausea and vomiting with female gender (rho = 0.196, *p* < 0.05), younger age (rho = −0.201, *p* < 0.05), and living in urban areas (rho = −0.190, *p* < 0.05); and dyspnea with older age (rho = −0.267, *p* < 0.05) and the presence of more comorbidities (rho = 0.232, *p* < 0.05).

In the context of multiple linear regression (Table 6) and using parameters that showed significant relationships based on univariate analysis, the increased global health status of the EORTC QLQ C30 was not significantly related to the characteristics of the patients (*p* > 0.05).

### 3.5. Correlations Between Symptoms and Patients’ Characteristics

The increase in the severity of the core symptoms of the MDASI was related to the female sex (rho = 0.193, *p* < 0.05) or the presence of more concomitant diseases (rho = 0.220, *p* < 0.05). Also, the total average symptom score seems to increase as the presence of more concomitant diseases increases (rho = 0.179, *p* < 0.05) (Table 8).

## 4. Discussion

The current study investigated the QOL and the symptoms of hospitalized patients with hematological malignancies. The results showed that patients with hematological malignancies have worse QOL, and there is a strong correlation between QOL and the symptoms of the disease and its treatments.

In the present study, the EORTC-QLQ-C30 assessment found that the cognitive function had the highest and the role function had the lowest levels. The QOL of 400 blood cancer patients in Pakistan was assessed using the EORTC-QLQ-C30. The highest scores were observed in the physical function and the lowest in the emotional function, followed by the cognitive function. Women and those patients treated in private sector healthcare facilities and distant and rural settings had relatively better QOL [17]. Also, physical problems (fatigue, dyspnea) are the most widely reported among patients with myelodysplastic syndromes, and then social function and role function are significantly impaired. Among these patients, worse QOL has been correlated with female gender, older age, poor performance status, more comorbidities, and sleep disturbances [18]. In addition, in a study of 68 Turkish patients with Hodgkin lymphoma, role function had the highest scores, while emotional function had the lowest [19]. Altogether, the QOL of patients with hematological malignancy was significantly worse in comparison with the general population (*p* < 0.001) but similar when compared with solid tumors and other chronic disabling diseases and lower than that due to multiple sclerosis (*p* = 0.032) [20].

The results of our research showed that the most aggravating symptoms for patients with hematological malignancies were pain and fatigue, which gradually brought restrictions to their daily activities. It is worth mentioning that the physical needs of patients with hematological malignancy are one of the most frequently mentioned categories of unsatisfied supportive care needs [9]. With the increasing research and the variety of available treatments for hematological malignancies, these diseases have turned into chronic diseases with a very high symptom burden [21].

Similar results to our study were reported in a study in Australia, evaluating symptoms with MDASI in 180 patients and concluding that the main symptom was fatigue (69%) and the least reported was vomiting (9%) [22]. Patients had a significant physical and psychological burden of symptoms, with a total average of 8.8 (±5.9) symptoms, and generally had low levels of QOL. In addition, in a study in Malaysia with 105 hospitalized patients with hematological malignancy, the four most common symptoms identified were fatigue, financial difficulties, reduced role function, and decreased social function [23]. Likewise, 110 inpatients and outpatients with non-Hodgkin lymphoma from seven hospitals in Ankara, Turkey, mainly reported fatigue, hair loss, and taste changes [24].

Moreover, 65% of our sample had sleep problems (drowsiness, insomnia) based on the MDASI. In a study in Japan with 153 hospitalized patients with hematological malignancies, insomnia was found in 60% [25]. Sleep problems are among the five most common symptoms affecting leukemia patients [26]. In hematological cancer patients, sleep problems are often coupled with higher levels of fatigue because they tend to leave patients without adequate energy to move and result in fatigue [27,28].Τhe problem is that a vicious cycle is caused since bad sleep causes the other symptoms to intensify (e.g., fatigue, anxiety, depression, pain), as well as, vice versa, these symptoms cause inadequate sleep [27], thus impairing the daily activities and QoL of patients. It is well acknowledged that all kinds of cancer patients often suffer from many physical, psychosocial, and mental problems, including sleep problems, especially insomnia. The prevalence of insomnia in cancer patients has been reported to be 50% or more, which is much higher in the general population (10.2–28.5%) [29].

In general, though, patients with solid tumors face the same symptoms. The most frequent side effects of chemotherapy reported by 153 Greek cancer patients using the MDASI were fatigue, nausea, constipation, anorexia, vomiting, pain, sadness, and anxiety [30]. Alamanou et al. (2016), when assessing 211 Greek cancer patients using the MDASI, found that the most intense primary symptoms were sadness, fatigue, sleep disorders, and anxiety, while the less intense symptoms were vomiting, nausea, and diarrhea [31].

Regarding the correlation of demographics with symptoms and QOL, the symptoms of the disease and the side effects of treatment have a more significant impact on QOL in women than in men. Women had an overall average symptom score higher than men. In contrast, in the study of La Nasa et al. (2020), there did not appear to be a significant difference between the two genders regarding their QOL [20]. Another study of 115 leukemia patients in Iran showed that there was a significant correlation between physical function with gender, educational level, and marital status, as well as a significant correlation between fatigue and pain [32]. Also, in Northwestern Turkey, researchers evaluated 332 patients with hematologic malignancies, and gender was not found to be associated with QOL or symptoms; only older age had a negative effect on physical function (as seen in our study). Their results are very much in line with our research. The highest average symptom score was fatigue, followed by pain, insomnia, and loss of appetite, ending with nausea and vomiting. Additionally, 73.1% of patients had reduced financial function, 39% had reduced physical function, 28.7% had reduced role and social function, 24.5% had reduced emotional function, and 15.4% had reduced cognitive function [33].

In addition, our results revealed that global health status was related to the male sex, while physical functioning was related to younger age, higher education, fewest months after diagnosis, and less comorbidity. Nausea and vomiting were also associated with the female gender, fatigue was associated with older age, and dyspnea with the presence of more comorbidity. The QOL of 131 survivors with hematological malignancy was assessed by Immanuel et al. (2019) using the EORTC QLQ-C30 questionnaire. Participants’ age was negatively correlated with global health status, physical functioning, and role functioning. Men had better physical functioning and reported fewer pain and sleep loss symptoms than women. The employed participants reported better physical, role, cognitive, and social functioning than the unemployed. On the other hand, the unemployed reported more fatigue, pain, dyspnea, sleep loss, appetite loss, and constipation compared with the employed ones [34].

Indeed, it is reported that modest notice has been given to the effect of hematological malignancies on patients’ QoL, even though it is acknowledged that patient experience, symptom burden, and social restrictions negatively influence their QOL and then their clinical outcomes [20,35]. The PROFILES disease-specific registry studies that were published in 2020 reported that non-Hodgkin lymphoma participants who had died had significantly lower EORTC QLQ-C30 total scores compared with those who were alive during follow-up. Also, significant associations between the EORTC QLQ-C30 total score and all-cause mortality were observed for non-Hodgkin lymphoma, chronic lymphocytic leukemia, and multiple myeloma. The same outline was observed for global QoL and physical functioning, although global QoL was also significantly associated with all-cause mortality for patients with Hodgkin lymphoma [36].

So, the relevant literature comes from many countries and settings, and something that should be noted is the influence of culture on the reporting of cancer symptoms and patients’ needs. Attitudes, beliefs, and values associated with health and illness vary among ethnically diverse groups [37]. Culture encompasses the ideas, customs, social behavior, attitudes, and characteristics of a group and the influences on peoples’ cancer beliefs. These beliefs are then affected by added economic, social, and health-related determinants [38], also shaping the cancer symptom report. The symptom report is clearly connected to culture, so, inevitably, some of them may have been over-mentioned or under-reported [39]. For example, in some cultures, even cancer symptoms (e.g., pain) can grow stigma. For these reasons, cultural aspects are progressively being recognized as important determinants of cancer prevention and outcomes following cancer diagnosis and treatment [37].

In the Results section, it is highlighted that the QoL of hematological cancer patients is considerably decreased during anticancer treatments due to a noteworthy number of reported symptoms. Nevertheless, the limitations of the study include the short period that the study was conducted, the relatively small and heterogeneous sample, the mixed recruitment, including inpatients and outpatients, and the data collection from only two hospitals. It was also a cross-sectional study that did not allow for changes to be found over time. Furthermore, treatments and questionnaires were self-completed by patients, so they are subject to subjectivity.

## 5. Conclusions

Patients with hematological malignancies endure many treatments and have a variety of physical and psychosocial symptoms, the most common of which are pain and fatigue, which significantly affect their QOL. Half of the patients in our study had moderate or low global health status of QOL. Cognitive function had higher levels, and role function had lower levels. Also, QOL and, specifically, the global health status subscale were associated with highly reported symptoms.

This specific issue under study is complex and demands further investigation. Future multicenter and prospective studies will lead to more valid and generalizable results for the QOL and symptoms of hospitalized patients with hematological malignancies. Future studies in this area could also focus on specific types of hematological malignancies and patients with advanced disease who face even more challenging issues.

The present study could guide healthcare professionals to more easily identify high-risk patients for reduced QOL and increased symptoms, problems, and needs. A clear understanding of the specific issues that are most important to this group of patients, through systematic evaluation, will help identify their most relevant concerns during treatment and plan appropriate interventions to provide more personalized, high-quality care. The continuous investigation of the changing symptoms and needs of this group of patients will contribute to their successful coverage.

## Figures and Tables

**Table 1:** Descriptive characteristics of the 120 patients with hematological malignancies.

		N	%
Gender	men/women	69/51	57.5/42.5
Age (years)	Mean age ± (min–max)	65.6 ± 17.5 (18.7–94.3)	
	<60	41	34.2
	60–79	55	45.8
	80+	24	20.0
Μarital Status	Unmarried/Divorced/Widowed	38	31.7
	Married, Cohabitation	82	68.3
Education	Primary school	51	42.5
	Middle school	22	18.3
	High school	23	19.2
	University	24	20.0
Living alone	Yes	20	16.7
	Νo	100	83.3
Employment	Unemployed/Retired/Housekeeping	89	74.2
	Employees	25	20.8
	Freelancers/Farmers	6	5.0
Residence	Urban area	86	71.7
	Rural Area	34	28.3
Time from diagnosis (months)	Mean (median) (min–max)	33 (48) (1–276)	
Comorbidities	Νone	44	36.7
	1	40	33.3
	2+	36	30.0

**Table 2:** Descriptive characteristics of EORTC QLQ-C30.

EORTC QLQ C30 Subscales	Mean	SD *	Median	Range
Global health status/QoL	47.1	21.5	50.0	0–100
Functional scales (Higher score shows better QoL)				
Physical functioning	58.1	25.5	60.0	0–100
Role functioning	46.1	32.8	41.7	0–100
Emotional functioning	64.7	27.3	75.0	0–100
Cognitive functioning	72.8	28.2	83.3	0–100
Social functioning	58.9	31.3	66.7	0–100
Symptom scales/items (Lower score shows better QoL)				
Fatigue	59.1	25.6	66.7	0–100
Nausea and vomiting	11.0	21.4	0,0	0–100
Pain	34.9	31.5	33.3	0–100
Dyspnea	44.7	31.9	33.3	0–100
Insomnia	35.8	34.1	33.3	0–100
Appetite loss	33.3	34.6	33.3	0–100
Constipation	23.6	32.5	0.0	0–100
Diarrhea	15.6	28.6	0.0	0–100
Financial difficulties	28.6	32.4	33.3	0–100

* SD: standard deviation.

**Table 3:** Hierarchical classification of the intensity and frequency of symptoms of MDASI.

Symptom Items		Mean	SD *	Median	Min	Max	N	%
Part I ^1^	Fatigue	3.5	3.2	2.0	0.0	10.0	94	78.3
	Drowsiness	3.3	3.4	2.0	0.0	10.0	78	65.0
	Distress	2.8	3.1	1.0	0.0	10.0	79	65.8
	Dry mouth	2.8	3.3	1.0	0.0	10.0	74	61.7
	Sadness	2.8	3.2	2.0	0.0	10.0	72	60.0
	Disturbed Sleep	2.5	3.0	1.0	0.0	10.0	72	60.0
	Anorexia	2.2	3.0	1.0	0.0	10.0	62	51.7
	Numbness and Tingling	2.1	3.1	0.0	0.0	10.0	52	43.3
	Pain	1.9	2.8	0.5	0.0	10.0	60	50.0
	Constipation	1.7	3.0	0.0	0.0	10.0	40	33.3
	Memory problems	1.6	2.7	0.0	0.0	10.0	51	42.5
	Shortness of breath	1.5	2.8	0.0	0.0	10.0	46	38.3
	Nausea	1.2	2.4	0.0	0.0	10.0	40	33.3
	Diarrhea	1.2	2.7	0.0	0.0	10.0	29	24.2
	Vomiting	0.6	1.7	0.0	0.0	10.0	19	15.8
Part II ^2^	Enjoyment of life	5.1	3.6	5.0	0.0	10.0	103	85.8
	Walking	4.7	3.5	4.0	0.0	10.0	99	82.5
	Mood	4.4	3.4	4.0	0.0	10.0	99	82.5
	Work	4.3	3.7	4.0	0.0	10.0	90	75.0
	General Activity	4.2	3.4	4.0	0.0	10.0	96	80.0
	Relations with other people	3.7	3.6	3.0	0.0	10.0	81	67.5

^1^ Response rating from 0: symptom did not present up to 10: the worst you can imagine. ^2^ Response rating from 0: symptom did not interfere to 10: symptom interfered completely. * SD: standard deviation.

**Table 4:** MDASI symptom intensity score.

MDASI	Mean	SD *	Median	Min	Max
Part I ^1^	2.1	2.0	1.5	0.0	10.0
Part II ^2^	4.4	3.1	4.7	0.0	10.0
Total Average Symptom Score	2.8	2.4	2.1	0.0	10.0

^1^ Response rating from 0: symptom did not present up to 10: the worst you can imagine. ^2^ Response rating from 0: symptom did not interfere to 10: symptom interfered completely. Mann–Whitney control among the two symptom groups, *p* < 0.001. * SD: standard deviation

**Table 5:** Correlations between EORTC QLQ C30 and MDASI.

Measurement of Symptom Intensity ^α^			
	Part I	Part II	Total Average Score
	rho-Spearman		
Global Health Status/QOL	−0.426 *	−0.500 *	−0.491 *
Functionality (higher score → better QOL)			
Physical	−0.380 *	−0.400 *	−0.418 *
Role	−0.257 *	−0.387 *	−0.337 *
Emotional	−0.560 *	−0.458 *	−0.549 *
Cognitive	−0.600 *	−0.509 *	−0.596 *
Social	−0.402 *	−0.470 *	−0.471 *
Symptoms (lower score → better QOL)			
Fatigue	0.571 *	0.596 *	0.622 *
Nausea and vomiting	0.488 *	0.340 *	0.470 *
Pain	0.436 *	0.464 *	0.493 *
Dyspnea	0.406 *	0.356 *	0.406 *
Insomnia	0.355 *	0.238 *	0.332 *
Appetite loss	0.456 *	0.487 *	0.508 *
Constipation	0.378 *	0.321 *	0.389 *
Diarrhea	0.214 *	0.247 *	0.256 *
Financial difficulties	0.245 *	0.193 *	0.253 *

^α^ Highest scores (→10) indicated higher symptom intensity. * *p* < 0.05.

**Table 6:** Multiple linear regression of the the global health status of the EORTC QLQ C30, patients’ characteristics, and the total average symptom score of the MDASI.

	Global Health Status/QOL (Higher Score → Better QOL)			
Prognostic Factors	Unstandardized Factor β	95% CI		p-Value
Gender (1:men, 2:women)	−4.03	−11.02	2.95	0.255
Age (years)	−0.08	−0.31	0.16	0.519
Marital status (1:Unmarried/Divorced/Widowed, 2:Married, Cohabitation)	2.11	−6.70	10.93	0.636
Εducation (1:Primary school, 2:Middle school, 3:High schoolο, 4:University)	1.68	−1.79	5.16	0.338
Living alone (1:yes, 2:no)	−0.23	−11.23	10.76	0.967
Residence (1:urban, 2:rural)	4.68	−3.45	12.80	0.257
Time from diagnosis (months)	−0.05	−0.12	0.02	0.191
Comorbidities (diseases)	−0.40	−3.96	3.16	0.825
Total Average Symptom Score MDASI	−4.91	−6.56	−3.26	<0.001
R^2^ (R^2^ adjusted)	0.341 (0.287)			

**Table 7:** Correlations between EORTC QLQ C30 and patients’ characteristics.

	Gender	Age	Marital Status	Education	Living Alone	Residence	Time Since Diagnosis	Comorbidities
	rho-Spearman							
Global Health Status/QOL	−0.185 *	−0.145	0.057	0.176	−0.051	0.092	−0.059	−0.160
Functionality (higher score → better QOL)								
Physical	−0.096	−0.331 *	0.052	0.239 *	−0.019	0.009	−0.199 *	−0.209 *
Role	0.002	−0.232 *	0.030	0. 099	−0.031	−0.010	0.016	0.011
Emotional	−0.150	−0.192 *	-0.029	0.143	−0.089	0.011	−0.019	−0.284 *
Cognitive	−0.004	−0.176	-0.052	0.062	−0.098	−0.071	−0.197 *	−0.173
Social	0.012	−0.167	-0.029	0.124	−0.099	−0.081	−0.199 *	−0.098
Symptoms (lower score → better QOL)								
Fatigue	0.057	0.277 *	0.131	−0.102	0.020	−0.060	0.080	0.162
Nausea and vomiting	0.196 *	−0.201 *	0.023	0.036	0.059	−0.190 *	−0.137	0.094
Pain	0.114	0.073	−0.085	−0.167	0.016	−0.061	0.115	0.192 *
Dyspnea	0.009	0.267 *	0.027	−0.175	−0.043	0.016	0.103	0.232 *
Insomnia	0.136	−0.008	0.085	0.003	0.014	−0.087	0.000	0.165
Appetite loss	0.034	0.083	0.068	0.050	0.084	−0.169	−0.066	0.130
Constipation	0.160	−0.052	−0.008	0.038	0.038	−0.132	−0.227 *	0.153
Diarrhea	−0.001	0.129	−0.023	−0.120	0.138	0.004	0.079	0.167
Financial difficulties	0.011	0.091	−0.168	−0.081	0.015	−0.021	0.237 *	0.113

* *p*-value < 0.05.

**Table 8:** Correlations between MDASI and patients’ characteristics.

	Gender	Age	Marital Status	Education	Living Alone	Residence	Time Since Diagnosis	Comorbidities
	rho-Spearman							
Part I	0.193 *	0.088	0.062	−0.154	0.150	−0.063	0.109	0.220 *
Part II	0.080	0.145	−0.015	−0.131	0.156	−0.054	0.036	0.085
Total Average Symptom Score	0.163	0.108	0.005	−0.134	0.156	−0.075	0.076	0.179 *

* *p*-value < 0.05.

## Data Availability

The data presented in this study are available on request from the corresponding author.

## References

[1] WhoQol Group (1998). Development of the World Health Organization WHOQOL-BREF quality of life assessment. Psychol. Med..

[2] Cella D.F. (1994). Quality of life: Concepts and definition. J. Pain Symptom Manag..

[3] Post M. (2014). Definitions of quality of life: What has happened and how to move on. Top. Spinal Cord Inj. Rehabil..

[4] Goerling U., Stickel A. (2014). Quality of life in oncology. Psycho-Oncol..

[5] Allart-Vorelli P., Porro B., Baguet F., Michel A., Cousson-Gélie F. (2015). Haematological cancer and quality of life: A systematic literature review. Blood Cancer J..

[6] Hall A., Lynagh M., Bryant J., Sanson-Fisher R. (2013). Supportive care needs of hematological cancer survivors: A critical review of the literature. Crit. Rev. Oncol. Hematol..

[7] Herrmann A., Mansfield E., Tzelepis F., Lynagh M., Hall A. (2020). Use of the supportive care framework to explore haematological cancer survivors’ unmet needs: A qualitative study. BMC Health Serv. Res..

[8] Papathanasiou I.V., Kelepouris K., Valari C., Papagiannis D., Tzavella F., Kourkouta L., Tsaras K., Fradelos E.C. (2020). Depression, anxiety and stress among patients with hematological malignancies and the association with quality of life: A cross-sectional study. Med. Pharm. Rep..

[9] Tsatsou I., Konstantinidis T., Kalemikerakis I., Adamakidou T., Vlachou E., Govina O. (2020). Unmet Supportive Care Needs of Patients with Hematological Malignancies: A Systematic Review. Asia Pac. J. Oncol. Nurs..

[10] Konstantinidis T., Philalithis A. (2014). Supportive care needs of advanced cancer patients. The nursing perspective. Arch. Hell. Med..

[11] Cella D., Stone A.A. (2015). Health-related quality of life measurement in oncology: Advances and opportunities. Am. Psychol..

[12] Di Maio M., Basch E., Denis F., Fallowfield L.J., Ganz P.A., Howell D., Kowalski C., Perrone F., Stover A.M., Sundaresan P. (2022). The role of patient-reported outcome measures in the continuum of cancer clinical care: ESMO Clinical Practice Guideline. Ann. Oncol..

[13] Apostolaki E., Tsagkaraki E., Chasouraki E. (2022). Evaluation of the Quality of Life and the Symptoms of Hospitalized Hematologically Patients. Bachelor’s Thesis.

[14] Aaronson N.K., Ahmedzai S., Bergman B., Bullinger M., Cull A., Duez N.J., Filiberti A., Flechtner H., Fleishman S.B., de Haes J.C. (1993). The European Organization for Research and Treatment of Cancer QLQ-C30: A quality-of-life instrument for use in international clinical trials in oncology. J. Natl. Cancer Inst..

[15] Mystakidou K., Tsilika E., Parpa E., Kalaidopoulou O., Smyrniotis V., Vlahos L. (2001). The EORTC core quality of life questionnaire (QLQ-C30, version 3.0) in terminally ill cancer patients under palliative care: Validity and reliability in a Hellenic sample. Int. J. Cancer.

[16] Mystakidou K., Cleeland C., Tsilika E., Katsouda E., Primikiri A., Parpa E., Vlahos L., Mendoza T., Greek M.D. (2004). Anderson Symptom Inventory: Validation and utility in cancer patients. Oncology.

[17] Malik M., Rizwan I., Hussain A. (2021). Health Related Quality of Life among Blood Cancer Patients in Pakistan: A Cross Sectional Survey. Inquiry.

[18] Oliva E.N., Platzbecker U., Fenaux P., Garcia-Manero G., LeBlanc T.W., Patel B.J., Kubasch A.S., Sekeres M.A. (2021). Targeting health-related quality of life in patients with myelodysplastic syndromes–Current knowledge and lessons to be learned. Blood Rev..

[19] Gemici A., Serin İ., Erol V.B., Doğu M.H., İnce İ., Eren R., Tekinalp A., Karakuş V., Sevindik Ö.G. (2022). Quality of Life assessment with EORTC QLQ in patients with hodgkin lymphoma: Multicenter study. Acta Oncol. Turc..

[20] La Nasa G., Caocci G., Morelli E., Massa E., Farci A., Deiana L., Pintus E., Scartozzi M., Sancassiani F. (2020). Health Related Quality of Life in Patients with Onco-hematological Diseases. Clin. Pract. Epidemiol. Ment. Health.

[21] Charles S.C., Loretta A.W. (2014). Symptom burden in hematologic malignancies. Blood.

[22] Manitta V., Zordan R., Cole-Sinclair M., Nandurkar H., Philip J. (2011). The symptom burden of patients with hematological malignancy: A cross-sectional observational study. J. Pain Symptom Manag..

[23] Priscilla D., Hamidin A., Azhar M.Z., Noorjan K.O., Salmiah M.S., Bahariah K. (2011). Quality of life among patients with hematological cancer in a Malaysian hospital. Med. J. Malays..

[24] Bolukbas F., Kutluturkan S. (2014). Symptoms and symptom clusters in non Hodgkin’s lymphoma patients in Turkey. Asian Pac. J. Cancer Prev..

[25] Tanimukai H., Hirai K., Adachi H., Kishi A. (2014). Sleep problems and psychological distress in family members of patients with hematological malignancies in the Japanese population. Ann. Hematol..

[26] Williams L.A., Garcia Gonzalez A.G., Ault P., Mendoza T.R., Sailors M.L., Williams J.L., Huang F., Nazha A., Kantarjian H.M., Cleeland C.S. (2013). Measuring the symptom burden associated with the treatment of chronic myeloid leukemia. Blood.

[27] Castelli L., Elter T., Wolf F., Watson M., Schenk A., Steindorf K., Bloch W., Hallek M., Joisten N., Zimmer P. (2022). Sleep problems and their interaction with physical activity and fatigue in hematological cancer patients during onset of high dose chemotherapy. Support. Care Cancer.

[28] Miladinia M., Baraz S., Ramezani M., Malehi A.S. (2018). The relationship between pain, fatigue, sleep disorders and quality of life in adult patients with acute leukaemia: During the first year after diagnosis. Eur. J. Cancer Care.

[29] Davidson J.R., MacLean A.W., Brundage M.D., Schulze K. (2002). Sleep disturbance in cancer patients. Soc. Sci. Med..

[30] Polikandrioti M., Gerasimou E., Kotronoulas G., Tsami A., Evagelou E., Kyritsi E. (2010). Evaluation of the Side-Effects of Chemotherapy in Patients with Cancer. Nosileftiki.

[31] Alamanou D., Ioannidou A., Poulianou E. (2016). Assessment of Symptoms in Cancer Patients. Nosileftiki.

[32] Musarezaie A., Khaledi F., Esfahani H.N., Ghaleghasemi T.M. (2014). Factors affecting quality of life and fatigue in patients with leukemia under chemotherapy. J. Educ. Health Promot..

[33] Pamuk G.E., Uyanik M.S., Harmandar F., Demir M. (2012). Health-related quality of life in hematological malignancy patients in northwestern turkey. Blood.

[34] Immanuel A., Hunt J., McCarthy H., van Teijlingen E., Sheppard Z.A. (2019). Quality of life in survivors of adult haematological malignancy. Eur. J. Cancer Care.

[35] Howell D.A., McCaughan D., Smith A.G., Patmore R., Roman E. (2022). Incurable but treatable: Understanding, uncertainty and impact in chronic blood cancers—A qualitative study from the UK’s Haematological Malignancy Research Network. PLoS ONE.

[36] Husson O., de Rooij B.H., Kieffer J., Oerlemans S., Mols F., Aaronson N.K., van der Graaf W.T.A., van de Poll-Franse L.V. (2020). The EORTC QLQ-C30 Summary Score as Prognostic Factor for Survival of Patients with Cancer in the “Real-World”: Results from the Population-Based PROFILES Registry. Oncologist.

[37] Daher M. (2012). Cultural beliefs and values in cancer patients. Ann. Oncol..

[38] Dein S. (2004). Explanatory models of and attitudes towards cancer in different cultures. Lancet Oncol..

[39] Guidry J.J., Torrence W., Herbelin S. (2005). Closing the divide: Diverse populations and cancer survivorship. Cancer.

